# Feller Processes: The Next Generation in Modeling. Brownian Motion, Lévy Processes and Beyond

**DOI:** 10.1371/journal.pone.0015102

**Published:** 2010-12-03

**Authors:** Björn Böttcher

**Affiliations:** TU Dresden, Institut für Mathematische Stochastik, Dresden, Germany; Università del Piemonte Orientale, Italy

## Abstract

We present a simple construction method for Feller processes and a framework for the generation of sample paths of Feller processes. The construction is based on state space dependent mixing of Lévy processes. Brownian Motion is one of the most frequently used continuous time Markov processes in applications. In recent years also Lévy processes, of which Brownian Motion is a special case, have become increasingly popular. Lévy processes are spatially homogeneous, but empirical data often suggest the use of spatially inhomogeneous processes. Thus it seems necessary to go to the next level of generalization: Feller processes. These include Lévy processes and in particular Brownian motion as special cases but allow spatial inhomogeneities. Many properties of Feller processes are known, but proving the very existence is, in general, very technical. Moreover, an applicable framework for the generation of sample paths of a Feller process was missing. We explain, with practitioners in mind, how to overcome both of these obstacles. In particular our simulation technique allows to apply Monte Carlo methods to Feller processes.

## Introduction

The paper is written especially for practitioners and applied scientists. It is based on two recent papers in stochastic analysis [1], [2]. We will start with a survey of applications of Feller processes. Thereafter we recall some existence and approximation results. In the last part of the introduction we give the necessary definitions.

The main part of the paper contains a simple existence result for Feller processes and a description of the general simulation scheme. These results will be followed by several examples.

The source code for the simulations can be found as supporting information (Appendix S1).

### Motivation

Brownian motion and more general Lévy processes are used as models in many areas: For example in medicine to model the spreading of diseases [3], in genetics in connection with the maximal segmental score [4], in biology for the movement patterns of various animals (cf. [5] and the references therein), for various phenomena in physics [6] and in financial mathematics [7]. In these models the spatial homogeneity is often assumed for simplicity, but empirical data or theoretical considerations suggest that the underlying process is actually state space dependent. Thus Feller processes would serve as more realistic models. We give some explicit examples:

- In hydrology stable processes are used as models for the movement of particles in contaminated ground water. It has been shown that state space dependent models provide a better fit to empirical data [8], [9]. Also on an intuitive level it seems natural that different kinds of soils have different properties. Thus the movement of a particle should depend on its current position, i.e. the soil it is currently in.
- In geology also stable processes are used in models for the temperature change. Based on ice-core data the temperatures in the last-glacial and Holocene periods are recorded. Statistical analysis showed that the temperature change in the last-glacial periods is stable with index 1.75 and in the Holocene periods it is Gaussian, i.e. stable with index 2 (see Fig. 4 in [10]).
- For a technical example from physics note that the fluctuations of the ion saturation current measured by Langmuir probes in the edge plasma of the Uragan-3M stellarator-torsatron are alpha-stable and the alpha depends on the distance from the plasma boundary [11].
- Anomalous diffusive behavior has been observed in various physical systems and a standard model for this behavior are continuous time random walks (CTRWs) [12], [13]. To study these systems the limiting particle distribution is a major tool, which is in fact a Feller process [14].
- In mathematical finance the idea of extending Lévy processes to Lévy-like Feller processes was first introduced in [15]. The proposed procedure is simple: A given Lévy model usually uses a parameter dependent class of Lévy processes. Now one makes the parameters of the Lévy process (in its characteristic exponent) *price*-dependent, i.e. the increment of the process shall depend on the current *price*. This procedure is applicable to every class of Lévy processes, but the existence has to be shown for each class separately [15]–[17].

Thus there is plenty of evidence that Feller processes can be used as suitable models for real-world phenomena.

### Existence and Approximation

Up to now general Feller processes were not very popular in applications. This might be due to the fact that the existence and construction of Feller processes is a major problem. There are many approaches: Using the Hille-Yosida theorem and Kolmogorov's construction [18], [19], solving the associated evolution equation (Kolmogorov's backwards equation) [20]–[23], proving the well-posedness of the martingale problem [16], [19], [24], solving a stochastic differential equation [25]–[27]. The conditions for these constructions are usually quite technical. Nevertheless, let us stress that the proof of the very existence is crucial for the use of Feller processes. Some explicit examples to illustrate this will be given at the end of the next section.

Our construction will not yield processes as general as the previous ones, but it will still provide a rich class of examples. In fact the presented method is just a simple consequence of a recent result on the solutions to certain stochastic differential equations [2].

Furthermore each of the above mentioned methods also provides an approximation to the constructed Feller process. Most of them are not usable for simulations or work only under technical conditions. Also further general approximation schemes exist, for example the Markov chain approximation in [28]. But also the latter is not useful for simulations, since the explicit distribution of the increments of the chain is unknown.

In contrast to these we derived in [1] a very general approximation scheme for Feller processes which is also usable for simulations. We will present here this method for practitioners.

### Lévy processes and Feller processes

Within different fields the terms *Lévy process* and *Feller process* are sometimes used for different objects. Thus we will clarify our notion by giving precise definitions and mentioning some of the common uses of these terms.

A stochastic process is a family of random variables indexed by a time parameter 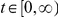 on a probability space 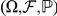. For simplicity we concentrate on one-dimensional processes. The expectation with respect to the measure 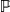 will be denoted by 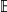.

Although this will not appear explicitly in the sequel, a process will always be equipped with its so-called natural filtration, which is a formal way of taking into account all the information related to the history of the process. Technically the filtration, which is an increasing family of sigma fields indexed by time, is important since a change from the natural filtration to another filtration might alter the properties of the process dramatically.

A **Lévy process** starting in 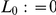 is a stochastic process 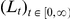 with

- independent increments: The random variables 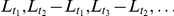 are independent for every increasing sequence 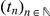,

- stationary increments: 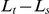 has the same distribution as 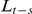 for all 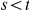,

- càdlàg paths: Almost every sample path is a right continuous function with left limits.

For equivalent definitions and a comprehensive mathematical treatment of Lévy processes and their properties see [29].

Note that the term *Lévy flight* often refers to a process which is a continuous time random walk (CTRW) with spatial increments from a one-sided or two-sided stable distribution (the former is also called Lévy distribution). In our notion the processes associated with these increments are Lévy processes which are called *stable subordinator* and *stable process*, respectively.

A Lévy process 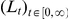 on its probability space is completely characterized by its Lévy exponent 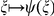 calculated via the characteristic function
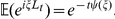



The most popular Lévy process is Brownian motion (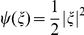), which has the special property that almost every sample path is continuous. In general, Lévy processes have discontinuous sample paths, some examples with their corresponding exponents are the Poisson process (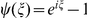), the symmetric 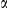-stable process (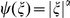 with 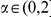), the Gamma process (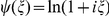) and the normal inverse Gaussian process (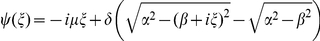 with 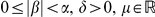).

Classes of Lévy exponents depend, especially in modeling, on some parameters. Thus one can easily construct a family of Lévy processes by replacing these parameters by state space dependent functions. Another approach to construct families of Lévy processes is to introduce a state space dependent mixing of some given Lévy processes. We will elaborate this in the next section.

Given a family of Lévy processes 
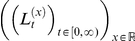
, i.e. given a family of characteristic exponents 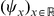, we can construct for fixed 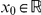, 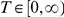, 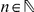 a Markov chain as follows:

1. The chain starts at time 0 in 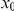.
2. The first step is at time 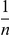 and it is distributed as 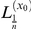. The chain reaches some point 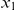.
3. The second step is at time 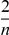 and it is distributed as 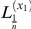. The chain reaches some point 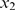.
4. The third step is at time 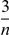 and it is distributed as 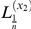. The chain reaches some point 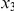.
5. etc. until time 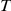.

This Markov chain is spatially inhomogeneous since the distribution of the next step always depends on the current position. If the chain converges (in distribution for 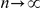 and every fixed 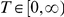) then the limit is - under very mild conditions (see [30] and also Theorem 2.5 by [31]) - a Feller process. Formally, a **Feller process** is a stochastic process 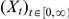 such that the operators
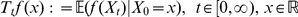



satisfy
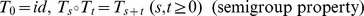



and
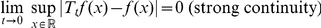



for all 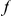 which are continuous and vanish at infinity.

A Feller processes is sometimes also called: Lévy-type process, jump-diffusion, process generated by a pseudo-differential operator, process with a Lévy generator or process with a Lévy-type operator as generator. Note that in mathematical finance often the Cox-Ingersoll-Ross process [32] is called *the Feller process*, but in our notion this is a Feller diffusion in the sense of [33]. For a comprehensive mathematical treatment of Feller processes and their properties see [18].

The generator 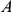 of a Feller process is defined via
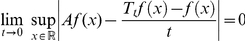
for all 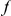 such that the limit exists. Moreover, if the limit exists for arbitrarily often differentiable functions with compact support then the operator 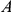 has on these functions the representation
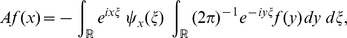
where for each fixed 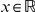 the function 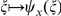 is a Lévy exponent. Thus a family of Lévy processes with Lévy exponents 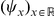 corresponds to the Feller process 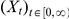 with generator 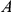 as above.

If the corresponding family of Lévy processes is a subset of a *named* class of Lévy processes, one calls the Feller process also by the name of the class and adds *-like* or *-type* to it. Thus for example a Feller process corresponding to a class of symmetric stable processes is called symmetric stable-like process.

In general, as mentioned in the previous section, the construction of a Feller process corresponding to a given family of Lévy processes is very complicated. It even might be impossible as the following examples show: Let 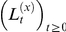 be the family of Lévy processes with characteristic exponents 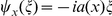, i.e. the Lévy processes have deterministic paths 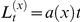. Now if 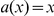 a corresponding Feller process exists, starting in 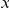 it has the path 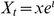. But for 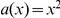 and 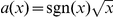 a corresponding Feller process does not exist, 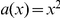 yields paths which do not tend to negative infinity as 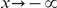 and 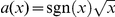 yields paths which are not continuous with respect to the starting position.

However, we will present in the next section a very simple method to construct Feller processes.

## Results and Discussion

### Construction of Feller processes by mixing Lévy processes

Suppose we know (for example based on an empirical study) that the process we want to model behaves like a Lévy process 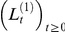 in a region 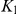 and like a different Lévy process 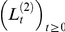 in a region 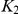. Then we know that a Feller process which models this behavior exists by the following result:

#### Theorem

If the sets 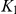, 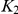 are uniformly separated, i.e. there exists an 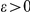 such that
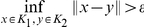
hen there exists a Feller process 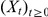 which behaves like 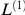 on 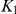 and like 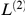 on 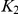.

#### Proof

Let 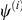 be the characteristic exponent of 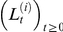 for 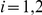. Under the above condition there exist non-negative bounded and Lipschitz continuous functions 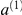 and 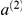 such that
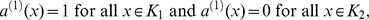


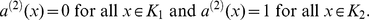



Now set for 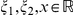


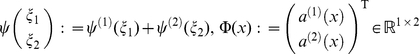
and note that for 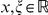


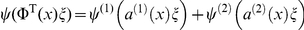
holds. Thus corresponding to the family of Lévy processes defined by the Lévy exponents
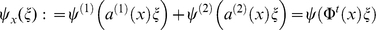
there exists a Feller process as a consequence of Corollary 5.2 from [2] and 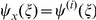 for 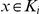 holds (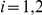), i.e. 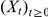 behaves like 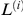 on 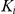 for 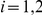.

Note that the theorem extends to any finite number of Lévy processes 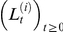 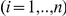 with corresponding regions 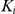. More generally for any finite number of independent Lévy processes 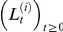 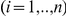 with corresponding characteristic exponents 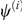 and non-negative bounded and Lipschitz continuous functions 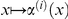 the family 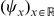 with
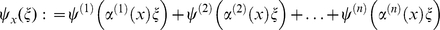
defines a family of Lévy processes 
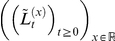
 and there exists a corresponding Feller process 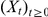.

To avoid pathological cases one should assume 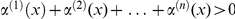 for all 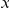. Further note that the following equality in distribution holds for all 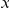 and 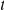


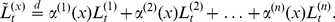



Thus if one knows how to simulate increments of the 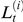 one can also simulate increments of 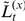. We will see in the next section that simulation of increments of the corresponding family of Lévy processes is the key to the simulation of the Feller process.

### Simulation of Feller processes

Given a Feller process 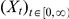 with corresponding family of Lévy processes 
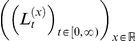
 we can use the following scheme to approximate the sample path of 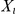:

1. Select a starting point 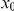, the time interval 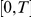 and the time-step size 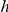.
2. The first point of the sample path is 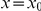 at time 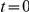.
3. Draw a random number 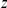 from the distribution of 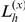 (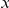 is the *current* position of the sample path).
4. The next point of the sample path is *x 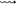 x + z* at time *t 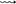 t + h*.
5. Repeat 3. and 4. until 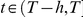.

The simulated path is an approximation of the sample path of the Feller process, in the sense that for 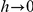 it converges toward the sample path of the Feller process on 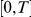. To be precise, for the convergence the Feller process has to be unique for its generator restricted to the test functions and the family of Lévy processes 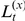 has to satisfy some mild condition on the 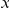-dependence: The Lévy exponent 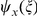 has to be bounded by some constant times 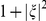 uniformly in 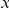, see [1] for further details. This condition is satisfied for many common examples of Feller processes, in particular for the processes constructed in the previous section.

The reader familiar with the Euler scheme for Brownian or Lévy-driven stochastic differential equations (SDEs) will note that the approximation looks like an Euler scheme for an SDE. In fact it is an Euler scheme, but the corresponding SDE does not have such a nice form as for example the Lévy-driven SDEs discussed in [34]. This is due to the fact that in their case for a particular increment all jumps of the driving term are transformed in the same manner, but in the general Feller case the transformation of each jump can depend explicitly on the jump size. More details on the relation of this scheme to an Euler scheme can be found in a forthcoming paper [35].

### Examples

We will now present some examples of Feller processes together with simulations of their sample paths. The first example will show the generality of the mixture approach, the remaining examples are special cases for which the existence has been shown by different techniques.

All simulations are done with the software package R [36] and the source code of the figures can be found as supporting information (Appendix S1).

### Brownian-Poisson-Cauchy-mixture Feller process

To show the range of possibilities which are covered by the mixture approach we construct a process which behaves like
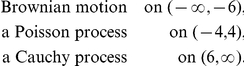



For this we just define a family of Lévy processes by the family of characteristic exponents 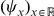 with
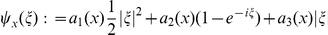
where
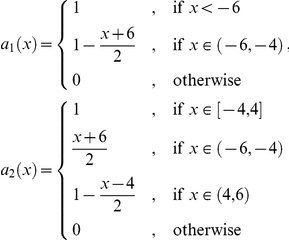
and
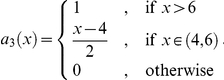



**Figure 1:**
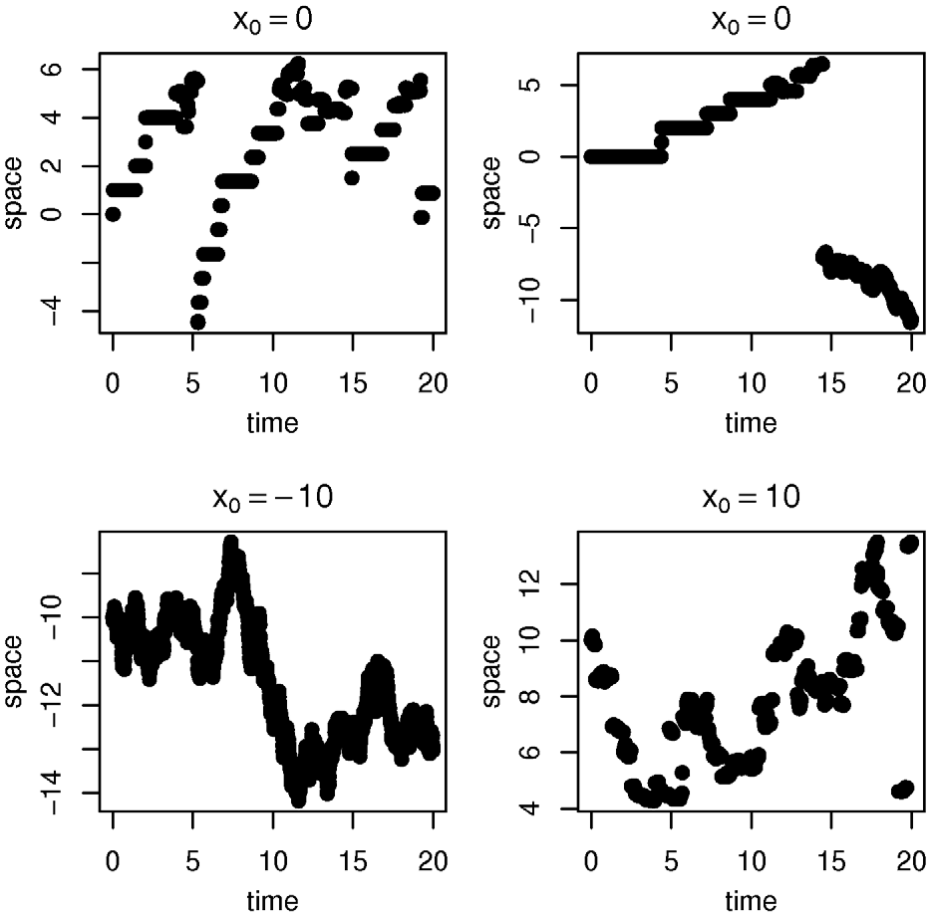
Brownian-Poisson-Cauchy-mixture Feller process. Around the origin it behaves like a Poisson process, for smaller values like Brownian motion and a for larger values like a Cauchy process.

These functions are Lipschitz continuous and thus a corresponding Feller process exists. Figure 1 shows some samples of this process on 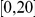 with time-step size 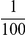. One can observe that the process behaves like a Poisson process around the origin, like a Cauchy process above 6 and like Brownian motion below −6.

### Symmetric stable-like process

A Lévy process 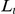 is a symmetric-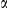-stable process if there exists an 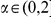 such that its characteristic function is given by
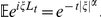



If we now define a function 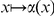 where 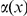 takes only values in 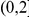 then there exists a family of of Lévy processes 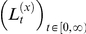 such that for fixed 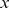 the the process 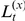 has the characteristic function
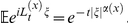



A corresponding Feller process exists and is unique if the function 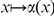 is Lipschitz continuous and bounded away from 0 and 2 [16].

**Figure 2:**
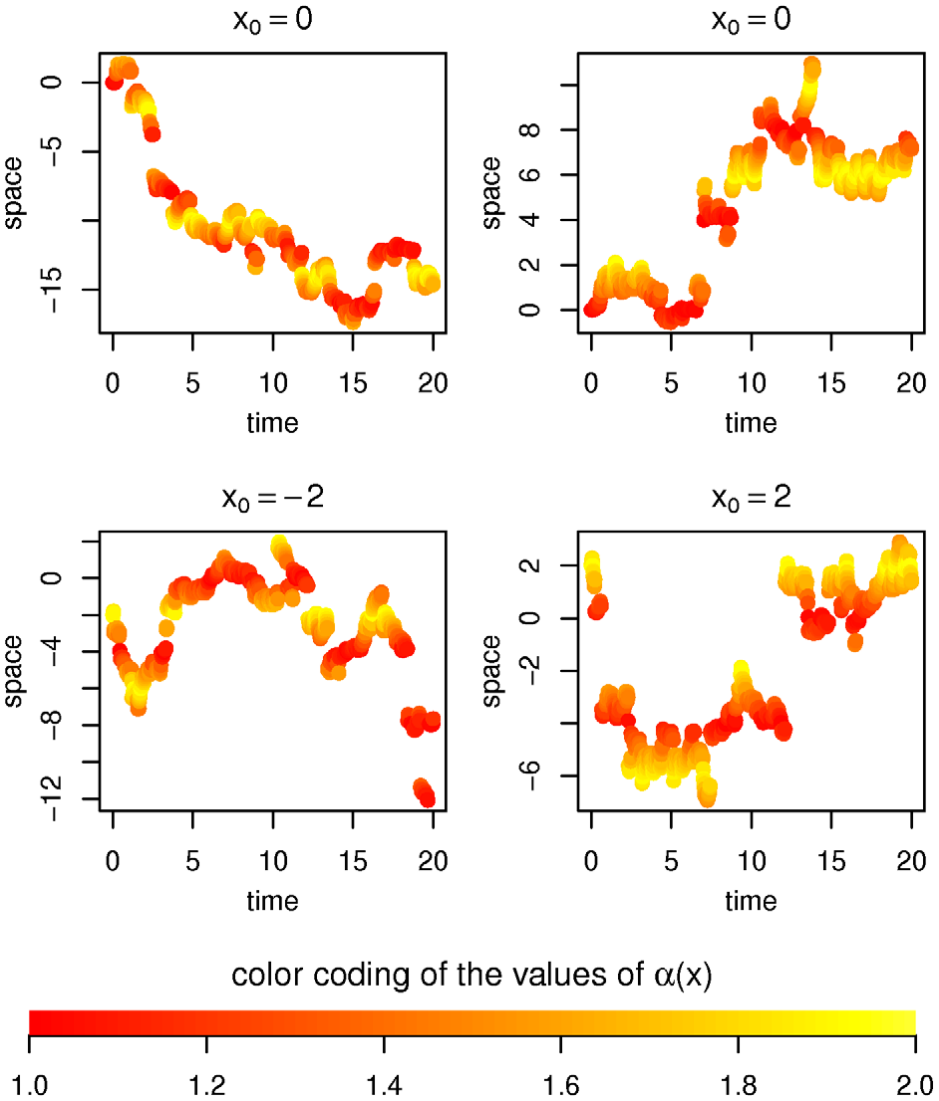
Stable-like processes with 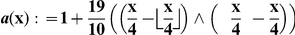. Each position is color coded by the corresponding value of the exponent. In the yellow regions it behaves almost like Brownian motion, in the red regions it behaves almost like a Cauchy process.

Figure 2 shows some samples of a stable-like Feller process on 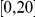 with time-step size 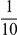 and
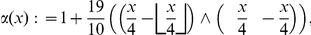
i.e. 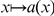 is a function which is Lipschitz continuous (but not smooth) oscillating between 1 and (nearly) 2. To understand the figure note that we color coded the state space: red indicates 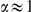, yellow indicates 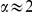 and the values between these extremes are colored with the corresponding shade of orange. Now one can observe that the process behaves in the red areas like a Cauchy process and the more *yellow* the state becomes, the more the process behaves like Brownian motion.

### Normal inverse Gaussian-like process

The characteristic function of a normal inverse Gaussian process 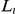 is given by
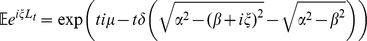
where 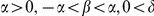 and 
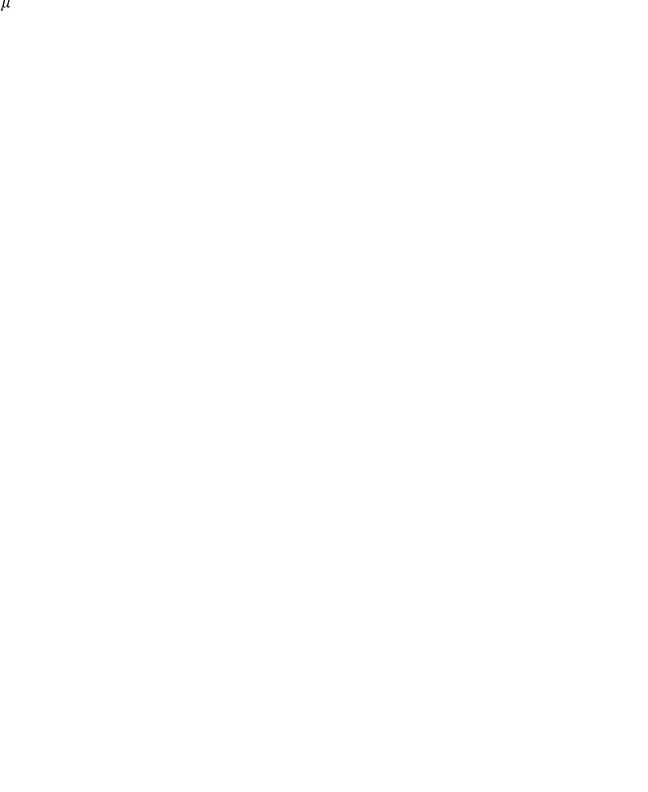
. If we replace 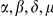 by arbitrarily often differentiable bounded functions 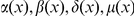 with bounded derivatives and assume that ther exist constants 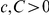 such that 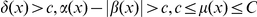, then it was stated in [15] that a corresponding Feller process exists. Therein was also proposed an example of a mean reverting normal inverse Gaussian-like process, a special case of this model with mean 0 is obtained by setting
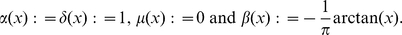



**Figure 3:**
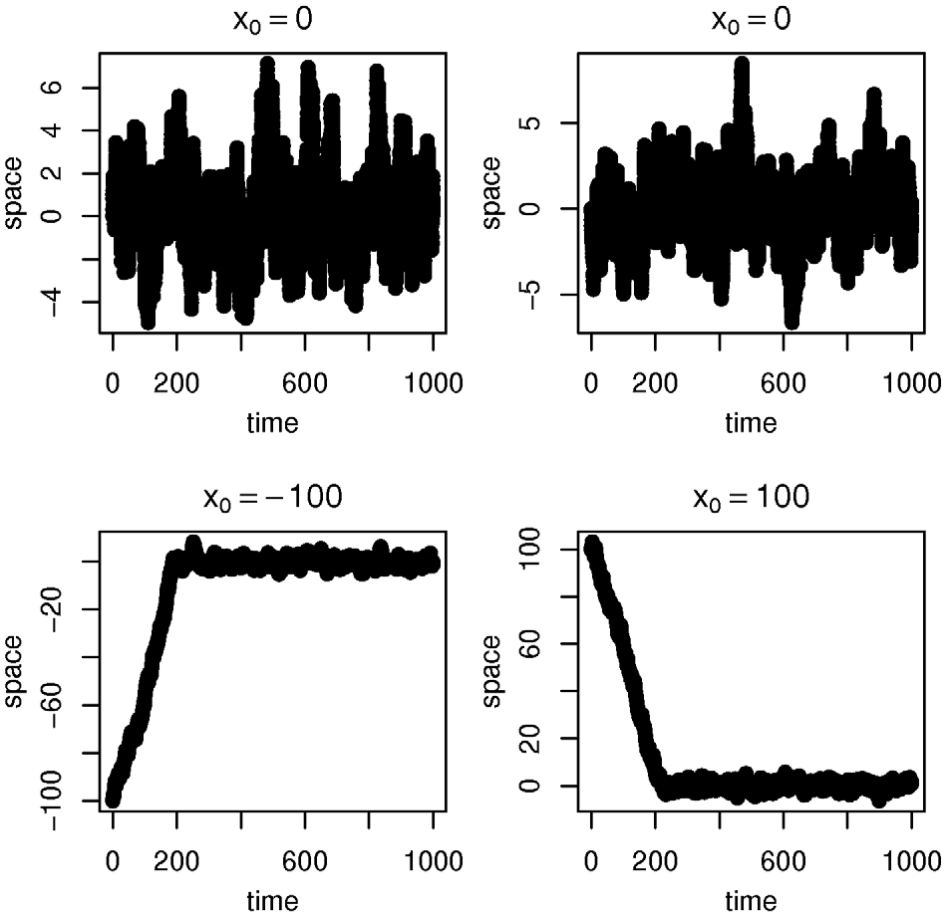
Normal inverse Gaussian-like processes with 
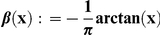
. The process features mean reversion to 0.

Note that the mean reversion is not introduced by using simply a drift which drags the process back to the origin. It is the choice of 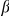 which yields an asymmetric distribution that moves the process back to the origin. The mean reversion can be observed in Figure 3 which shows samples of the normal inverse Gaussian-like process on 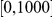 with time-step size 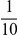.

### Meixner-like process

The characteristic function of a Meixner process 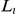 is given by
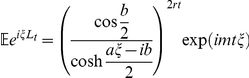
where 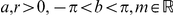. Details can be found in [37].

A family of Meixner processes which corresponds to a Feller process can be constructed by substituting the parameters 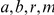 by arbitrary often differentiable bounded functions 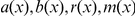 with bounded derivatives. The functions have to be bounded away from the critical values, i.e. 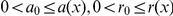 and 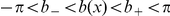 for some fixed 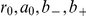. For further details see [17].

**Figure 4:**
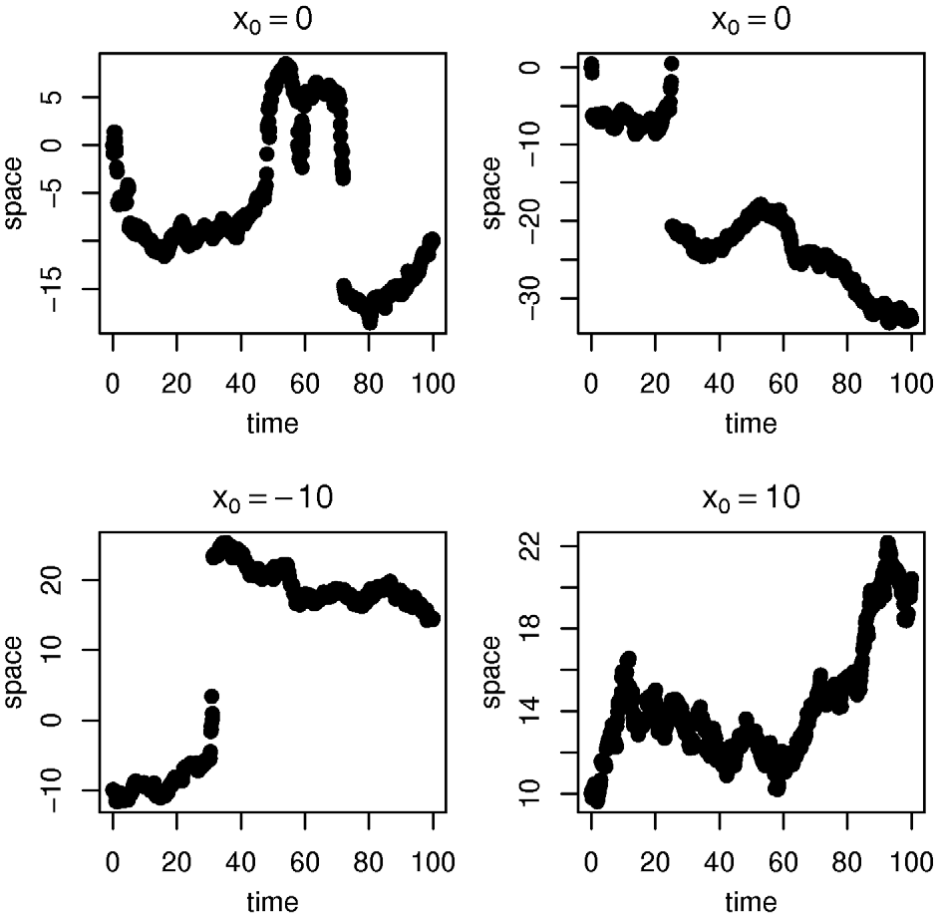
Meixner-like process with 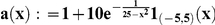. The process moves *with bigger steps* around the origin than for larger (and smaller) values. In fact by the choice of 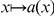 the rate of the exponential decay of the transition density is reduced around the origin.

Figure 4 shows some samples of the Meixner-like process on 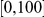 with time-step size 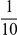 and
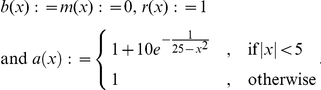



The chosen functions satisfy the existence conditions from above. Furthermore the function 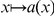 yields that the process *moves with bigger steps* around the origin, to be precise: the Meixner distribution has semiheavy tails [38] and the parameter 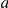 determines the rate of the exponential decay factor for the density. The effect on the sample path can be observed in Figure 4.

### Conclusion

Using the presented mixture approach one can easily construct Feller models based on given Lévy models. In these cases the existence of the process is granted.

Furthermore the presented approximation is a very intuitive way to generate the sample path of a Feller processes. Obviously the method requires that one can simulate the increments of the corresponding Lévy processes. But for Lévy processes used in applications, especially together with Monte Carlo techniques, this poses no new restriction.

Thus all necessary tools are available to use Feller processes as models for a wide range of applications.

## Materials and Methods

The simulations where done in R [36] and the source code of the figures can be found as supporting information (Appendix S1).

## Supporting Information

Appendix S1 Source code of the figures.
(TXT)

## References

[1] Böttcher B, Schilling R (2009). Approximation of Feller processes by Markov chains with Lévy increments.. Stoch Dyn.

[2] Schilling R, Schnurr A (2010). The Symbol Associated with the Solution of a Stochastic Differential Equation.. Electron J Probab.

[3] Janssen H, Oerding K, van Wijland F, Hilhorst H (1999). Lévy-flight spreading of epidemic processes leading to percolating clusters.. Eur Phys J B.

[4] Doney RA, Maller RA (2005). Cramér's Estimate for a Reflected Lévy Process.. Ann Appl Probab.

[5] Reynolds AM FM (2007). Free-flight odor tracking in drosophila is consistent with an optimal intermittent scale-free search. 2(4): e354. doi:.. PLoS ONE.

[6] Woyczyński WA (2001). Lévy processes in the physical sciences.. Lévy Process-Theory and Applications, Birkhäuser.

[7] Cont R, Tankov P (2004). Financial modelling with jump processes.. Chapman & Hall/CRC.

[8] Zhang Y, Benson D, Meerschaert M, LaBolle EM (2007). Space-fractional advection-dispersion equations with variable parameters: Diverse formulas, numerical solutions, and application to the MADE-site data.. Water Resources Research.

[9] Reeves D, Benson D, Meerschaert M, Scheffler H (2008). Transport of Conservative Solutes in Simulated Fracture Networks 2. Ensemble Solute Transport and the Correspondence to Operator-Stable Limit Distributions.. Water Resources Research.

[10] Ditlevsen PD, Svensmark H, Johnsen S (1996). Contrasting atmospheric and climate dynamics of the last-glacial and Holocene periods.. Nature.

[11] Gonchar VY, Chechkin AV, Sorokovoi EL, Chechkin VV, Grigoreva LI (2003). Stable Lévy distributions of the density and potential fluctuations in the edge plasma of the U-3M torsatron.. Plasma Physics Reports.

[12] Meerschaert MM, Benson DA, Scheffler HP, Becker-Kern P (2002). Governing equations and solutions of anomalous random walk limits.. Physical Review E.

[13] Scalas E, Gorenflo R, Mainardi F (2004). Uncoupled continuous-time random walks: Solution and limiting behavior of the master equation.. Physical Review E.

[14] Baeumer B, Meerschaert M, Mortensen J (2005). Space-time fractional derivative operators.. Proc Amer Math Soc.

[15] Barndorff-Nielsen O, Levendorskiľ S (2001). Feller processes of normal inverse Gaussian type.. Quant Finance.

[16] Bass R (1988). Uniqueness in law for pure jump Markov processes.. Probab Theory and Relat Fields.

[17] Böttcher B, Jacob N (2003). Remarks on Meixner-type processes.. Probabilistic Methods in Fluids (eds IM Davies et al).

[18] Jacob N (2001). Pseudo-differential operators and Markov processes, volume I-III..

[19] Hoh W (1998). Pseudo differential operators generating Markov processes..

[20] Böttcher B (2008). Construction of time inhomogeneous Markov processes via evolution equations using pseudo-differential operators.. J Lon Math Soc.

[21] Böttcher B (2005). A parametrix construction for the fundamental solution of the evolution equation associated with a pseudo-differential operator generating a Markov process.. Math Nachr.

[22] Kochubei AN (1989). Parabolic pseudodifferential equations, hypersingular integrals and Markov processes.. Math USSR Izvestija.

[23] Kolokoltsov VN (2000). Symmetric stable laws and stable-like jump-diffusions.. Proc London Math Soc.

[24] Stroock D (1975). Diffusion processes associated with levy generators.. Probab Theory Relat Fields.

[25] Ikeda N, Watanabe S (1989). Stochastic Differential Equations and Diffusion Processes, volume 24 of *Math. Library*. North-Holland, 2nd edition.

[26] Jacod J, Shiryaev AN (2002). Limit Theorems for Stochastic Processes.. Springer, 2nd edition.

[27] Stroock DW (2003). Markov Processes from K. Itô's Perspective..

[28] Ma ZM, Röckner M, Zhang TS (1998). Approximation of arbitrary Dirichlet processes by Markov chains.. Ann Inst Henri Poincare.

[29] Sato K (1999). Lévy Processes and Infinitely Divisible Distributions..

[30] Chernoff P (1974). Product Semigroups, Nonlinear Semigroups and Addition of unbounded Operators.. Number 140 in Mem. AMS.

[31] Jacob N, Potrykus A (2005). Roth's method applied to some pseudo-differential operators with bounded symbols. A case study.. Rend Cir Mat Palermo (Ser II).

[32] Cox JC, Ingersoll JE, Ross SA (1985). A theory of the term structure of interest rates.. Econometrica.

[33] Feller W, Neyman J (1951). Diffusion processes in genetics.. Proceedings of the Second Berkeley Symposium on Mathematical Statistics and Probability.

[34] Protter P, Talay D (1997). The Euler scheme for Lévy driven stochastic differential equations.. Ann Probab.

[35] Böttcher B, Schnurr A (2010). The Euler scheme for Feller processes.. http://arxiv.org/abs/0911.5245.

[36] R Development Core Team (2010). R: A language and environment for statistical computing.. http://www.R-project.org.

[37] Grigelionis B (1999). Processes of Meixner type.. Lithuanian Math J.

[38] Grigelionis B (2001). Generalized *z*-distributions and related stochastic processes.. Lithuanian Math J.

